# Strain variation in the *Candida albicans* iron limitation response

**DOI:** 10.1128/msphere.00372-24

**Published:** 2024-07-09

**Authors:** Liping Xiong, Katharina Goerlich, Eunsoo Do, Aaron P. Mitchell

**Affiliations:** 1 Department of Microbiology, University of Georgia, Athens, Georgia, USA; University of Michigan Michigan Medicine, Ann Arbor, Michigan, USA

**Keywords:** *Candida albicans*, strain variation, iron homeostasis, DNA repair

## Abstract

Iron acquisition is critical for pathogens to proliferate during invasive infection, and the human fungal pathogen *Candida albicans* is no exception. The iron regulatory network, established in reference strain SC5314 and derivatives, includes the central player Sef1, a transcription factor that activates iron acquisition genes in response to iron limitation. Here, we explored potential variation in this network among five diverse *C. albicans* strains through mutant analysis, Nanostring gene expression profiling, and, for two strains, RNA-Seq. Our findings highlight four features that may inform future studies of natural variation and iron acquisition in this species. (i) Conformity: In all strains, major iron acquisition genes are upregulated during iron limitation, and a *sef1*Δ/Δ mutation impairs that response and growth during iron limitation. (ii) Response variation: Some aspects of the iron limitation response vary among strains, notably the activation of hypha-associated genes. As this gene set is tied to tissue damage and virulence, variation may impact the progression of infection. (iii) Genotype-phenotype variation: The impact of a *sef1*Δ/Δ mutation on cell wall integrity varies, and for the two strains examined the phenotype correlated with *sef1*Δ/Δ impact on several cell wall integrity genes. (iv) Phenotype discovery: DNA repair genes were induced modestly by iron limitation in *sef1*Δ/Δ mutants, with fold changes we would usually ignore. However, the response occurred in both strains tested and was reminiscent of a much stronger response described in *Cryptococcus neoformans*, a suggestion that it may have biological meaning. In fact, we observed that the iron limitation of a *sef1*Δ/Δ mutant caused recessive phenotypes to emerge at two heterozygous loci. Overall, our results show that a network that is critical for pathogen proliferation presents variation outside of its core functions.

**IMPORTANCE:**

A key virulence factor of *Candida albicans* is the ability to maintain iron homeostasis in the host where iron is scarce. We focused on a central iron regulator, *SEF1*. We found that iron regulator Sef1 is required for growth, cell wall integrity, and genome integrity during iron limitation. The novel aspect of this work is the characterization of strain variation in a circuit that is required for survival in the host and the connection of iron acquisition to genome integrity in *C. albicans*.

## INTRODUCTION

*Candida albicans* is a prevalent human fungal pathogen and is included in the World Health Organization (WHO) fungal pathogen critical priority group. It is estimated that over a billion people worldwide develop *Candida* bloodstream or deep tissue infection per year, with over 60% mortality (1). There is a vital need to develop new antifungals (2), and these efforts are informed by the determinants of *C. albicans* growth and virulence.

*C. albicans*, like many pathogens, requires iron (3). Iron cofactors activate enzymes and stabilize protein structures in support of central cellular processes such as respiration, metabolism, translation, and DNA replication [reviewed in reference (4)]. *C. albicans* inhabits host niches that differ markedly in iron availability, from the iron-replete gastrointestinal tract to the iron-limited bloodstream. Iron acquisition and utilization by *C. albicans* are controlled by the transcription factors (TFs) Sef1, Sfu1, and Hap43 (5, 6). Sef1 is required for *C. albicans* growth in iron-depleted media and virulence in murine-invasive infection models (5, 6). In response to iron depletion, Sef1 is phosphorylated by the Ssn3 kinase and localized to the nucleus, where it activates the transcription of genes for the uptake of iron, siderophores, and heme (5, 6). Sef1 shares some target genes with two other TFs, the positive regulator Hap43 and the negative regulator Sfu1 (5). The balance of activities of these three TFs is critical for scavenging iron when it is scarce and avoiding toxicity of iron when it is abundant.

Much of our understanding of *C. albicans* regulatory mechanisms comes from studies of the clinical isolate SC5314 and its marked derivatives. However, *C. albicans* clinical isolates vary dramatically in growth and virulence-related phenotypes (7). Several advances in genetic methods have made it feasible to test gene function in diverse *C. albicans* isolates. There are multiple genome sequences (7, 8), CRISPR methods to accelerate recovery of homozygous mutants (9), an adjuvant strategy to improve drug marker phenotypes (10), and a CRISPR-based marker recycling strategy (11). In most species, including *C. albicans*, genotype-phenotype relationships can be highly variable among strains or individuals (7, 12–17). The advances in genetic methods enable us to see which determinants of *C. albicans* growth and virulence are most uniform across isolates, hence most promising for therapeutic targeting.

Here, we explored variation in iron regulation among a panel of diverse *C. albicans* isolates. What might we look for? Our interest has been shaped by prior studies of strain variation in biofilm and hyphal regulation (7, 12–17). What emerged from those studies were three features that may be manifested in other regulatory systems. First, the phenotypic impact of regulatory mutations can be highly variable among strains, both at the level of cellular or community behavior and at the level of gene expression outputs (7, 12–17). Second, while it may be difficult to identify causal mutations among strains, phenotypic differences can be explained by gene expression differences, which in turn can identify new regulatory interactions (13). Third, some differences in gene expression between a mutant and its respective wild type are uniform among strains; these conforming changes can reveal unanticipated functional relationships (17). Those three features may be paradigms reflected broadly or perhaps just novel attributes of the complex biofilm/hyphal regulatory architecture. Here, we initiate an analysis of the iron regulatory network in multiple *C. albicans* strains, a network that is critical for survival and may be constrained in variation.

## MATERIALS AND METHODS

### Media and culture conditions

Stock strains were maintained in 15% glycerol at −80°C. Streaked strains were maintained on Yeast extract Peptone Dextrose (YPD) agar plates (2% dextrose, 2% Bacto peptone, 1% yeast extract, and 2% Bacto Agar), and overnight cultures were grown in liquid YPD medium (2% dextrose, 2% Bacto peptone, and 1% yeast extract) at 30°C with rotation. Transformants were selected on synthetic Complete Supplement Mixture (CSM) minus histidine agar plates (2% dextrose, 0.67% Difco yeast nitrogen base w/o amino acids and ammonium sulfate, 0.077% MP CSM-Histidine, and 2% Bacto Agar) or selected for nourseothricin-resistance on YPD + 400 µg/mL nourseothricin (clonNAT, Gold Biotechnology). The strains used in this study are listed in Table S1.

To assay growth properties, SD medium (2% dextrose, 0.67% Difco yeast nitrogen base w/o amino acids) was adjusted to neutral pH (pH 7.0) with 25 mM HEPES buffer (Sigma-Aldrich H3375), and 400 µM of bathophenanthrolinedisulfonate (BPS; Sigma-Aldrich 11890) was added to achieve low-iron conditions. Sole iron sources including 100 µM of ferric chloride (Sigma-Aldrich 155740), 50 µM of hemin (Sigma-Aldrich H9039) (18), 20 µg/mL of hemoglobin (Sigma-Aldrich 08449) (19), 20 µg/mL of ferritin (Sigma-Aldrich F4503) (19), and 20 µg/mL of transferrin (Sigma-Aldrich T3309) (20) were added to the low-iron SD medium. To assay cell wall integrity, strains were grown on solid RPMI-1640 media (Sigma-Aldrich, Inc., St. Louis) with and without caspofungin (Sigma-Aldrich SML0425) or calcofluor white (Sigma-Aldrich F3543). We note that Roswell Park Memorial Institute 1640 Medium, which we refer to below as RPMI, is a low-iron medium (https://www.sigmaaldrich.com/US/en/technical-documents/technical-article/cell-culture-and-cell-culture-analysis/mammalian-cell-culture/media-formulations-rpmi-1640). To assay loss of heterozygosity (LOH) of *URA3* mutation, cell dilutions were plated on agar plates of CSM (synthetic SD medium with amino acid supplement) and CSM supplemented with 80 mg/L of uridine (Sigma-Aldrich U3003) and 1 g/L of 5-fluoroorotic acid (5-FOA; ZYMO RESEARCH F9001).

### Mutant construction

To manipulate the *C. albicans* genome, the transient CRISPR-Cas9 system was employed (9). Briefly, the Cas9 cassette was amplified from the plasmid pV1093 (21), and each sgRNA cassette was generated by using split-joint PCR with “sgRNA/F YFG1” and “SNR52/R YFG1” as previously described in detail (9, 12). PCR products were transformed into *C. albicans* cells using the lithium acetate transformation method (11). Homozygous mutants were constructed in *C. albicans* SC5314, P76067, P57055, P75010, and P87 background strains (7). Primers and plasmids used in this study are listed in Table S1.

To construct *sef1*Δ/Δ in the SC5314 strain background, two halves of *SEF1* deletion cassettes were amplified from the plasmid pmh01 with primers “HIS1 CRIME/F” and “SEF1_HIS1/AR,” and from the plasmid pmh02 with primers “SEF1_HIS1/AF” and “HIS1 CRIME/R,” respectively. Strain MC5 was transformed with approximately 1 µg of Cas9 DNA cassette, 1 µg of SEF1-sgRNA DNA cassette, 2 µg of *HIS1_01*, and 2 µg of *HIS1_02* repair template. Transformants were selected on CSM media lacking histidine. Candidate colonies were further genotyped by PCR using primers “SEF1_CK/F” and “SEF1_CK_Int/R” for the absence of the *SEF1* Open Reading Frame (ORF) and using primers “SEF1_CK/F” and “HIS1_CK_Int/R” for the presence of the *HIS1* marker at the *SEF1* locus.

To construct *sfu1*Δ/Δ in the SC5314 strain background, two halves of *SFU1* deletion cassettes were amplified from the plasmid pmh01 with primers “HIS1 CRIME/F” and “SFU1_HIS1/AR,” and from the plasmid pmh02 with primers “SFU1_HIS1/AF” and “HIS1 CRIME/R,” respectively. Strain MC5 was transformed with approximately 1 µg of Cas9 DNA cassette, 1 µg of SFU1-sgRNA DNA cassette, 2 µg of *HIS1_01*, and 2 µg of *HIS1_02* repair template. Transformants were selected on CSM media lacking histidine. Candidate colonies were further genotyped by PCR using primers “SFU1_CK/F” and “SFU1_CK_Int/R” for the absence of the *SFU1* ORF and using primers “SFU1_CK/F” and “HIS1_CK_Int/R” for the presence of the *HIS1* marker at the *SFU1* locus.

To construct *hap43*Δ/Δ in the SC5314 strain background, two halves of *HAP43* deletion cassettes were amplified from the plasmid pmh01 with primers “HIS1 CRIME/F” and “HAP43_HIS1/AR,” and from the plasmid pmh02 with primers “HAP43_HIS1/AF” and “HIS1 CRIME/R,” respectively. Strain MC5 was transformed with approximately 1 µg of Cas9 DNA cassette, 1 µg of HAP43-sgRNA DNA cassette, 2 µg of *HIS1_01*, and 2 µg of *HIS1_02* repair template. Transformants were selected on CSM media lacking histidine. Candidate colonies were further genotyped by PCR using primers “HAP43_CK_Int/F” and “HAP43_CK/R” for the absence of the *HAP43* ORF and using primers “HIS_CK/F” and “HAP43_CK/R” for the presence of the *HIS1* marker at the *HAP43* locus.

To construct *sef1*Δ/Δ, *sfu1*Δ/Δ, and *hap43*Δ/Δ in P76067, P57055, P75010, and P87 strain backgrounds, the transformation was conducted in strains MC1, MC2, MC4, and MC3, respectively, following with the genotyping same as mutant construction in the SC5314 strain background. The *NAT1* maker was recycled as described previously (12). To generate nourseothricin sensitive (NAT^s^) mutants, *sef1*Δ/Δ, *sfu1*Δ/Δ, and *hap43*Δ/Δ mutants were transformed with approximately 1 µg of Cas9 DNA cassette and 1 µg of NAT1-5 sgRNA DNA cassette. Transformants were plated on YPD agar plates. Candidate colonies were then streaked on YPD + NAT agar to screen for NAT^s^ colonies.

To construct ectopic expression strains, we replaced the *MDR1* ORF with wild-type *SEF1*, *SFU1*, or *Hap43* alleles using our concatemer assembly method (22). A cassette containing 1,645 bp of *SEF1* upstream sequence, the *SEF1* ORF, and 447 bp of *SEF1* downstream sequence was amplified from SC5314 genomic DNA using primers “SEF1 5’F-> MDR1 up/F” and “SEF1 3’R-> pNAT 5’/R,” containing concatenating homology to a NAT1 marker. A NAT1 marker was amplified from the plasmid pNAT using “pNAT/AF” and “pNAT 3’R - > MDR1 down/AR.” The transformation was conducted in *sef1*Δ/Δ NAT^s^ strains with approximately 1 µg of Cas9 DNA cassette, 1 µg of MDR1-5 sgRNA DNA cassette, 2 µg of *SEF1* cassette, and 2 µg of *NAT1* cassette. Transformants were screened on YPD plate containing 400 µg/mL nourseothricin, and candidate colonies were genotyped by PCR using primers “MDR1_CK_up/F” and “MDR1_CK_int/R” for the absence of *MDR1* ORF and using primers “MDR1_CK_up/F” and “SEF1_CK_int/R” for the presence of repair template at the *MDR1* locus.

To construct *SFU1* ectopic expression strains, a cassette containing 2,084 bp of *SFU1* upstream sequence, the *SFU1* ORF, and 488 bp of *SFU1* downstream sequence was amplified from SC5314 genomic DNA using primers “SFU1 5’F-> MDR1 up/F” and “SFU1 3’R-> pNAT 5’/R,” containing concatenating homology to a *NAT1* marker. A NAT1 marker was amplified from the plasmid pNAT using “pNAT/AF” and “pNAT 3’R - > MDR1 down/AR.” The transformation was conducted in *sfu1*Δ/Δ NAT^s^ strains with approximately 1 µg of Cas9 DNA cassette, 1 µg of MDR1-5 sgRNA DNA cassette, 2 µg of *SFU1* cassette, and 2 µg of *NAT1* cassette. Transformants were screened on YPD plate containing 400 µg/mL nourseothricin, and candidate colonies were genotyped by PCR using primers “MDR1_CK_up/F” and “MDR1_CK_int/R” for the absence of *MDR1* ORF and using primers “MDR1_CK_up/F” and “SFU1_CK_int/R” for the presence of repair template at the *MDR1* locus.

To construct *HAP43* ectopic expression strains, a cassette containing 1,521 bp of *HAP43* upstream sequence, the *HAP43* ORF, and 584 bp of *HAP43* downstream sequence was amplified from SC5314 genomic DNA using primers “*HAP43* 5’F-> MDR1 up/F” and “*HAP43* 3’R-> pNAT 5’/R,” containing concatenating homology to a NAT1 marker. A NAT1 marker was amplified from the plasmid pNAT using “pNAT/AF” and “pNAT 3’R - > MDR1 down/AR.” The transformation was conducted in *hap43*Δ/Δ NAT^s^ strains with approximately 1 µg of Cas9 DNA cassette, 1 µg of MDR1-5 sgRNA DNA cassette, 2 µg of *HAP43* cassette, and 2 µg of *NAT1* cassette. Transformants were screened on YPD plate containing 400 µg/mL nourseothricin, and candidate colonies were genotyped by PCR using primers “MDR1_CK_up/F” and “MDR1_CK_int/R” for the absence of *MDR1* ORF and using primers “MDR1_CK_up/F” and “HAP43_CK_int/R” for the presence of repair template at the *MDR1* locus.

To construct *ade2*Δ/*ADE2* heterozygous mutants in the wild-type and *sef1*Δ/Δ backgrounds, an *ADE2* deletion cassette was amplified from plasmid pNAT using primers “ADE2_pNAT/AF” and “ADE2_pNAT/AR.” Wild-type SC5314 and *sef1*Δ/Δ NAT^s^ strains were transformed with approximately 1 µg of Cas9 DNA cassette, 1 µg of ADE2-sgRNA DNA cassette, and 2 µg of *ADE2* deletion cassette. Transformants were screened on YPD plate containing 400 µg/mL nourseothricin, and candidate colonies were genotyped by PCR using primers “ADE2_CK_up/F” and “pANT_CK_int/R” for the presence of the *NAT1* marker at the *ADE2* locus and using primers “ADE2_CK_up/F” and “ADE2_CK_int/R” for the presence of *ADE2* ORF. Generally, *ade2*Δ/Δ mutants form unique red pigment, and *ade2*Δ/*ADE2* heterozygous mutants show normal white colony morphology.

To construct *ura3*Δ/*URA3* heterozygous mutants in the wild-type SC5314 and *sef1*Δ/Δ backgrounds, a *URA3* deletion cassette was amplified from plasmid pNAT using primers “URA3_pNAT/AF” and “URA3_pNAT/AR.” Wild-type SC5314 and *sef1*Δ/Δ NAT^s^ strains were transformed with approximately 1 µg of Cas9 DNA cassette, 1 µg of URA3-sgRNA DNA cassette, and 2 µg of *URA3* deletion cassette. Transformants were screened on YPD plate containing 400 µg/mL nourseothricin, and candidate colonies were genotyped by PCR using primers “URA3_CK_up/F” and “pANT_CK_int/R” for the presence of the 75010*NAT1* marker at the *URA3* locus and using primers “URA3_CK_up/F” and “URA3_CK_int/R” for the presence of *URA3* ORF.

### Spotting plate assays

Cells were harvested from overnight culture in SD and washed with H_2_O. Cells were then diluted in H_2_O to an OD_600_ of 3.0 measured with a spectrophotometer. Five-fold dilutions were spotted using a multichannel pipette on the indicated medium. Plates were incubated for 3 days at 37°C as indicated.

### RNA extraction and RNA-Seq

For RNA sample preparation, cells were grown in 5 mL of liquid YPD rotating at 75 rpm at 30°C overnight. The next day, cells were inoculated to 25 mL of 37°C pre-warmed medium YPD with or without 500 µM of BPS at an OD_600_ of 0.2. Cells were then grown for 4 hours with vigorous shaking (225 rpm) in an incubator shaker then harvested by vacuum filtration and quickly frozen at −80°C until RNA extraction (23). Three biological replicates were provided for RNA-Seq experiments.

RNA extraction was performed according to previously published methods (23). Cell disruption was achieved mechanically using Zirconia beads (Ambion, Fisher Scientific, Waltham), and extraction was performed using a 25:24:1 phenol:chloroform:isoamyl alcohol method combined with a Qiagen RNeasy Mini Kit (Qiagen, Venlo, Netherlands). RNA-Seq and bioinformatic analysis were performed by Novogene.

### Nanostring RNA assay

Nanostring analysis was performed as previously described (24). Gene expression was measured using the nCounter SPRINT Profiler. For our analysis, 38 iron-responsive genes, 9 hypha-associated genes, a cell-surface adhesion gene *ALS1*, their regulatory gene *EFG1*, and 4 house-keeping genes (*ARP3*, *CDC28*, *FKH2*, and *GIN4*) (12) were selected for the code set. For each Nanostring assay, 15 or 30 ng of RNA was added to the Nanostring codeset mix and incubated at 65°C overnight (16–18 hours). The samples were loaded onto the cartridge according to the manufacturer’s instructions and placed in the instrument for scanning and data collection. Raw counts were normalized against average total counts with background subtraction. Statistical significance in differential expression was assessed using the Benjamini-Hochberg procedure at an False Discovery Rate (FDR) of 0.1.

### Loss of heterozygosity assays

To evaluate the frequency of loss of heterozygosity, *ade2*Δ/*ADE2* colonies were grown in 5 mL of liquid YPD at 30°C with rotation overnight. Cells were then inoculated to 5 mL of fresh YPD at an OD_600_ of 0.05 and then grew for 24 hours at 30°C with rotation at 75 rpm. The next day, cells were diluted in H_2_O to an OD_600_ of 3.0, and 100 µL of 10^−4^ dilution was plated on the indicated agar plates YPD and YPD + BPS_100 μM_, respectively. Plates were incubated at 37°C, and the colony-formation unit (CFU) was counted when colonies grew to the appropriate size. After 9-day incubation, colonies on YPD and YPD + BPS_100 μM_ agar plates were stamped to fresh YPD agar plates and grew at 30°C for another 5 days to 1 week. One colony with more than one sector was counted as one. Sector frequency is calculated as the ratio of number of colonies with sectors to the total number of CFUs.

In a second LOH assay, *ura3*Δ/*URA3* cells were grown and treated similarly as *ade2*Δ/*ADE2* cells. After being grown on agar plates YPD and YPD + BPS_100 μM_ at 37°C for 9 days, cells were harvested from the plates by washing and resuspended in H_2_O. Each sample was then diluted in H_2_O to an OD_600_ of 3.0 and 100 µL of 10-time serial dilutions from 10^0^ to 10^−4^ were spread on two agar plates: CSM and CSM + uridine + 5-FOA, respectively. Plates were incubated at 30°C. CFU formed on each plate was counted 2 days later. The ratio of a number of 5-FOA resistance colonies to the number of total colonies was normalized to per 1 × 10^6^ cells.

### Data analysis software and statistics

Single-guide RNA sequences were checked for specificity using Cas-OFFinder software (25). Analyses were performed with GraphPad Prism version 10 (Graphpad Software, Inc., La Jolla). Venn diagrams were constructed by Venn Diagrams software (http://bioinformatics.psb.ugent.be/webtools/Venn/). Gene ontology (GO) term enrichment was performed using the FungiFun online tool (https://elbe.hki-jena.de/fungifun/). To generate a cnet plot graph, we implemented clusterProfiler (v4.8.1) in R by creating a GO term library using FungiDB (Candida albicans.Eupath.v65) with the R AnnotationForge package (26). Genes were defined by having an adjusted *P*-value < 0.05 and a fold change on a log_2_ scale of >1. Only GO categories with a *P*-value < 0.05 were considered significant. Heatmaps were generated with MeV 4.9.0 software (https://sourceforge.net/projects/mev-tm4/files/mev-tm4/). Genome sequences, annotations, and phenotype information were retrieved from the Candida Genome Database (27) and FungiDB (28).

## RESULTS AND DISCUSSION

### Growth properties of iron homeostasis mutants

**Fig 1:**
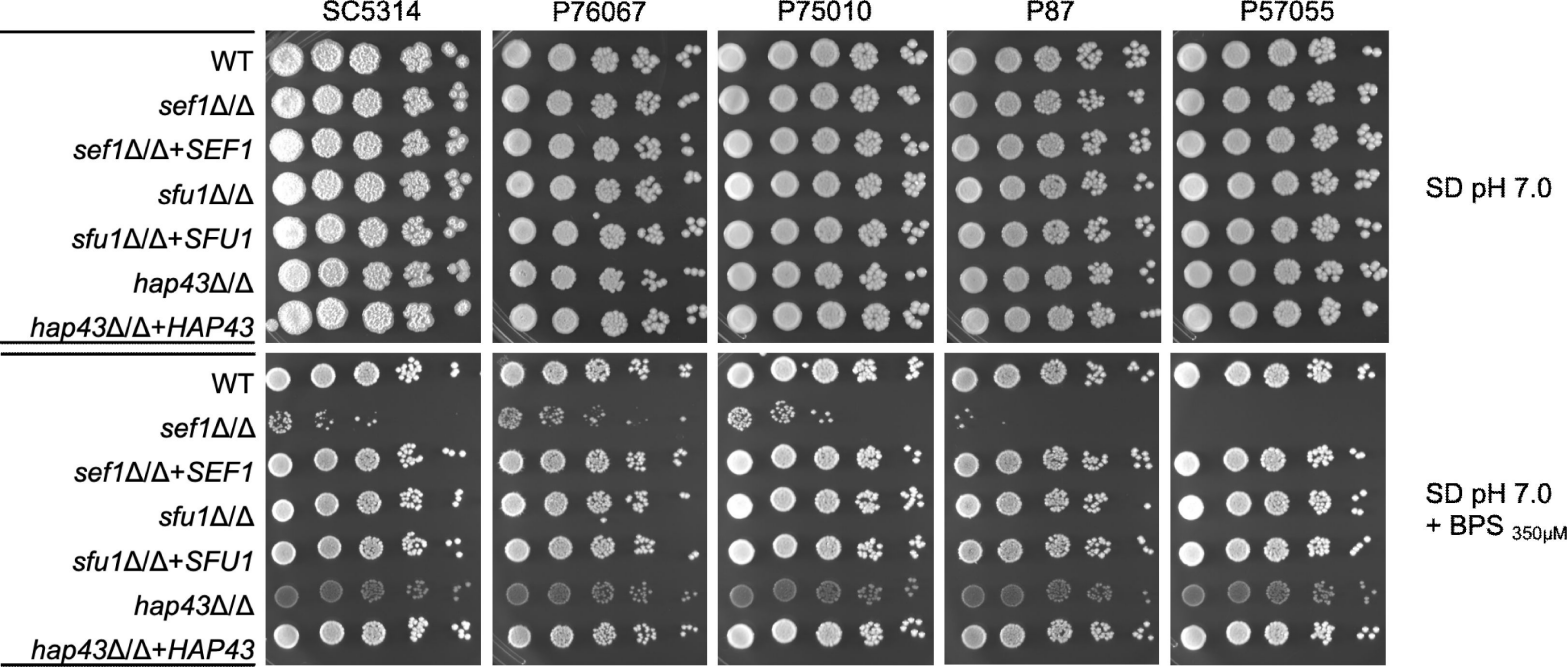
Growth property assays. Five wild-type strains of *C. albicans* (SC5314, P76067, P75010, P87, and P57055) and their respective *sef1*Δ/Δ*, sfu1*Δ/Δ, and *hap43*Δ/Δ derivatives were assayed for growth in SD pH 7.0 with and without 350 µM BPS. Precultures were grown in SD medium at 30°C for 16 hours with shaking. Cells were harvested, washed with H_2_O, and then diluted to OD_600_ ~ 3 in H_2_O. Serial dilutions of 5^−3^ to 5^−7^ were spotted in indicated agar plates. Plates were incubated at 37°C; images were taken at 72 hours.

We constructed *sef1*Δ/Δ, *sfu1*Δ/Δ, and *hap43*Δ/Δ mutants in five clinical isolates: SC5314 (clade 1), P76067 (clade 2), P57055 (clade 3), P87 (clade 4), and P75010 (clade 11) (7). Strains SC5314, P76067, P57055, and P75010 originate from bloodstream infections, and P87 originates from oral tissue (7). The mutants and their complemented strains were assayed for growth under iron-limiting conditions. We used SD (pH 7.0) plates with or without the iron chelator BPS (350 µM) at 37°C (Fig. 1). The conditions of neutral pH at 37°C were chosen to partially mimic the host environment. All strains tested grew similarly well on SD (pH 7.0) without BPS. In the presence of BPS, the wild-type and *sfu1*Δ/Δ mutant strains grew well; the *sef1*Δ/Δ mutants showed a pronounced growth defect; the *hap43*Δ/Δ mutants showed a mild growth defect and pink coloration. All mutant growth defects were rescued by complementation (Fig. 1). Overall growth properties of the mutants in all strains followed the same trend as found in prior studies of the SC5314 background (5, 29).

We observed some variation in the extent of *sef1*Δ/Δ mutant growth in the presence of BPS; there was less residual growth in P57055 and P87; there was more in SC5314, P76067, and P75010 (Fig. 1). A slight increase in the BPS concentration, to 400 µM, reduced residual growth in the SC5314 and P75010 backgrounds (Fig. S1). Hence residual growth probably reflects iron acquisition ability on BPS medium, rather than iron storage during prior growth.

The *hap43*Δ/Δ coloration phenotype has not been reported previously to our knowledge. We speculate that the red pigment may form via a glutathione-mediated detoxification pathway. In *Saccharomyces cerevisiae*, the monothiol glutaredoxin Grx4 is essential for the formation of red pigmentation (30). In *C. albicans*, the monothiol glutaredoxin Grx3 is required for growth on low iron, and it functionally interacts with Hap43 (31). Therefore, we hypothesize that the *hap43*Δ/Δ red pigmentation phenotype in low-iron media may reflect monothiol glutaredoxin accumulation.

We also tested the ability of these strains to utilize alternative iron sources, including hemin, hemoglobin, ferritin, and transferrin, on SD (pH 7.0) + BPS plates. Growth of *sef1*Δ/Δ mutants was greatly improved by the addition of hemin, moderately improved by the addition of hemoglobin, and unaffected by the addition of ferritin or transferrin (Fig. S1). Control plates with ferric chloride supplementation supported the strong growth of *sef1*Δ/Δ mutants (Fig. S1). Therefore, the impact of *sef1*Δ/Δ on the utilization of these alternative iron sources was generally uniform among strains.

**Fig 2:**
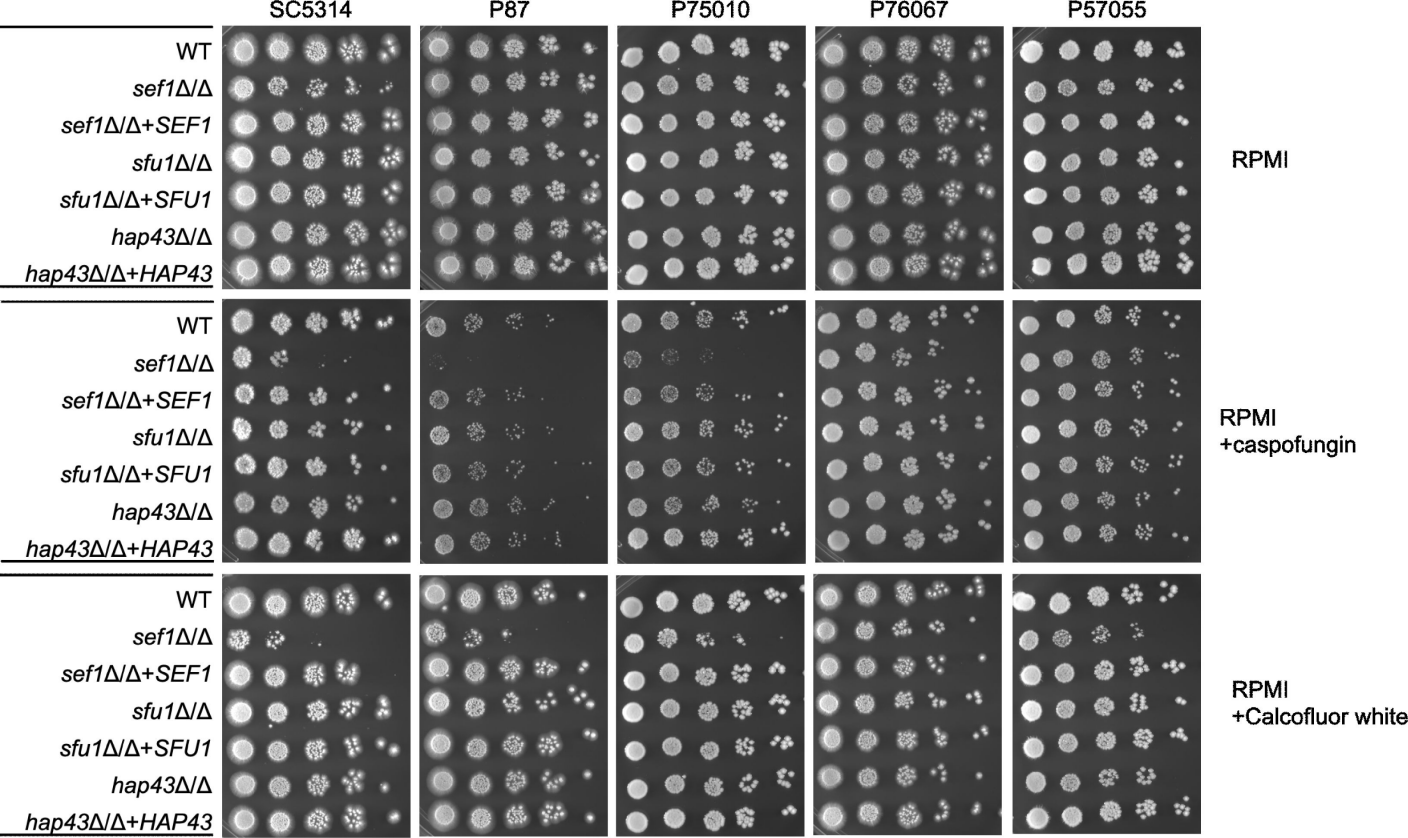
Cell wall integrity phenotypes. Five wild-type strains of *C. albicans* (SC5314, P87, P75010, P76067, and P57055) and their respective *sef1*Δ/Δ, *sfu1*Δ/Δ, and *hap43*Δ/Δ derivatives were assayed for cell wall integrity with caspofungin or calcofluor white in RPMI (pH 7.4) solid media. Precultures were grown in SD medium at 30°C for 16 hours with shaking. Cells were harvested, washed with H_2_O, and then diluted to OD_600_ ~ 3 in H_2_O. Serial dilutions of 5^−3^ to 5^−7^ were spotted in indicated agar plates. Caspofungin concentrations were 75 ng/mL (SC5314 and P75010 strain sets), 100 ng/mL (P76067 and P57055 strain sets), and 125 ng/mL (P87 strain set). Calcofluor white concentrations were 10 µM (SC5314 strain set) and 6 µM (P87, P75010, P76067, and P57055 strain sets). Plates were incubated at 37°C; images were taken at 72 hours.

A *sef1*Δ/Δ mutant made in strain SN152, a derivative of SC5314, was reported previously to be hypersensitive to the cell wall inhibitor caspofungin (32). Here, we tested that phenotype with growth on RPMI plates, a low-iron medium, with and without caspofungin at 37°C (Fig. 2). We observed that *sef1*Δ/Δ mutants of SC5314, P87, and P75010 showed greatly increased sensitivity to caspofungin; the *sef1*Δ/Δ mutant of P76067 showed mildly increased sensitivity; and the *sef1*Δ/Δ mutant of P57055 showed little sensitivity (Fig. 2). Tests with the cell wall perturbing agent calcofluor white trended similarly (Fig. 2). *SEF1* complementation rescued sensitivity (Fig. 2). We note that *sef1*Δ/Δ mutants showed no increased sensitivity to caspofungin on iron-replete YPD plates (Fig. S2). The *sfu1*Δ/Δ and *hap43*Δ/Δ mutants were not hypersensitive to either caspofungin or calcofluor white (Fig. 2). These results verify that *sef1*Δ/Δ mutations can cause increased sensitivity to cell wall perturbation, and strain background affects this phenotype.

### Focused gene expression profiling of iron homeostasis mutants

To determine the gene expression impact of *sef1*Δ/Δ, *sfu1*Δ/Δ, and *hap43*Δ/Δ mutations across our strain set, we conducted Nanostring gene expression profiling. The probe set (Table S2) was designed to detect RNA from the three TF genes (*SEF1*, *HAP43*, and *SFU1*), 36 Sef1 direct target genes (5), 9 hypha-associated genes, cell-surface adhesin gene *ALS1*, hyphal regulatory gene *EFG1* (17), and 4 control probes for normalization (12). Pilot studies indicated that YPD-based media supported a larger range of expression of Sef1 target genes than SD- or RPMI-based media. Therefore, we selected YPD medium at 37°C as the iron-replete condition and YPD + BPS medium at 37°C as the iron-limited condition for our assays.

**Fig 3:**
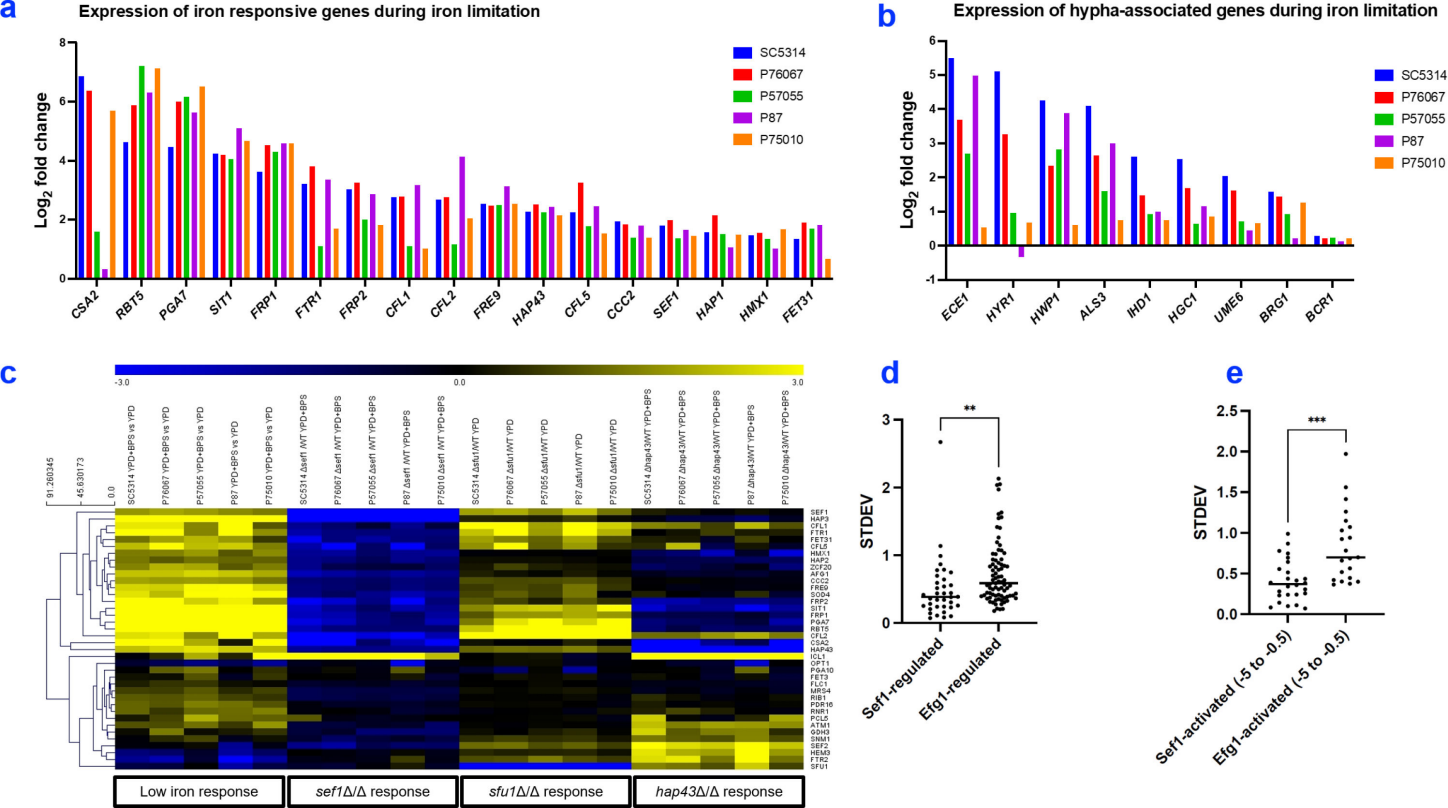
Nanostring profiling of iron-regulated genes. (a) Expression of Sef1 target genes under iron limitation in five *C. albicans* isolates. Each bar represents the average expression change of each gene based on its Log_2_ fold change of triplicates in YPD + BPS vs YPD of indicated strain and probe gene. (b) Expression of hypha-associated genes during iron limitation in five *C. albicans* isolates. Again, each bar represents the average expression change of each gene based on its Log_2_ fold change of triplicates in YPD + BPS vs YPD of indicated strain and probe gene. (c) Heatmap representation of Nanostring gene expression data. Strains were grown in the indicated media at 37°C for 4 hours with shaking. Nanostring probes included three TF genes (*SEF1*, *HAP43*, and *SFU1*) and 36 known Sef1 direct target genes (Table S2). The low-iron response was assayed with wild-type strains grown in YPD + BPS vs YPD. The *sef1*Δ/Δ response was assayed with *sef1*Δ/Δ mutants vs their respective wild types grown in YPD + BPS. The *sfu1*Δ/Δ response was assayed with *sfu1*Δ/Δ mutants vs their respective wild types grown in YPD. The *hap43*Δ/Δ response was assayed with *hap43*Δ/Δ mutants vs their respective wild types grown in YPD + BPS. Yellow color indicates increased expression, blue color indicates reduced expression, and black color indicates unchanged expression, with a numerical scale in Log_2_ fold change. (d) Scatter plot graph of SD. The SD of expression changes in *sef1*Δ/Δ vs wild-type comparisons (Table S2, tab E) or in published *efg1*Δ/Δ vs wild-type comparisons (12) was determined for the five strains SC5314, P76067, P57055, P87, and P75010, using Log_2_ fold change values. We sampled 36 Sef1 direct targets or 81 Efg1 direct targets, all assayed by Nanostring. Each dot represents the SD calculated for one gene based on its Log_2_ fold change among five strains. (e) Scatter plot graph of SD for select induced target genes. The SD of expression changes in *sef1*Δ/Δ vs wild-type comparisons or in published *efg1*Δ/Δ vs wild-type comparisons (12) was determined as described in panel d, but only genes with a Log_2_ fold change between −5 and −0.5 were included. This constraint reduced the sample size to 26 Sef1 direct targets and 21 Efg1 direct targets. For panels (d) and (e), statistical analysis was performed using a *t*-test . ***P-value < 0.001 and **P-value < 0.01.

We first assayed the low-iron response with wild-type strains grown in YPD + BPS vs YPD. YPD + BPS growth caused the upregulation of iron acquisition genes (e.g., *RBT5*, *PGA7*, and *SIT1*) in all strains (Fig. 3a; Table S2). The one exception was *CSA2*, which was not upregulated in strain P87 (Fig. 3a; Table S2). YPD + BPS growth also caused the upregulation of hypha-associated genes (e.g., *ECE1*, *HWP1*, and *ALS3*) in all strains except P75010 (Fig. 3b; Table S2). We note that strain P75010 produces hyphae weakly under many different growth conditions, and the overall extent of upregulation correlated with biofilm formation ability (Fig. S3), which depends upon hypha formation. The upregulation of hypha-associated genes during iron limitation had not been observed by Chen et al. (5), probably because they used a growth temperature of 30°C rather than 37°C. These results show that iron acquisition genes are induced by iron limitation uniformly among strains, whereas hypha-associated genes are induced variably.

We assayed the impact of Sef1 by comparing each *sef1*Δ/Δ mutant vs its respective wild-type strain, both grown in YPD + BPS (Fig. 3c; Table S2). The *sef1*Δ/Δ mutants all expressed the iron acquisition genes at lower levels than the respective wild-type strains. In contrast, the *sef1*Δ/Δ mutants caused little change in the expression of hypha-associated genes; the greatest impact was in strain SC5314 (Table S2). The iron acquisition genes assayed are direct Sef1 targets, and many are required for growth in low-iron media (33–38); their control by Sef1 is uniform among strains. The hypha-associated genes are not direct Sef1 targets and, with the exception of *ALS3* (19), have no known role in adaptation to low iron; they represent a Sef1-independent feature of the response to iron limitation.

We also assayed the impact of Hap43 in YPD + BPS (Fig. 3c). The *hap43*Δ/Δ mutants showed downregulation of a subset of iron acquisition genes, perhaps due to upregulation of their repressor Sfu1 (Fig. 3c; Table S2). All *hap43*Δ/Δ mutants showed upregulation of iron utilization genes (e.g., *ICL1*, *CFL2*, and *HEM3*; Fig. 3c; Table S2), as expected from studies in the SC5314 background (5). Also, *hap43*Δ/Δ mutations had variable effects on hypha-associated gene expression (Table S2).

The impact of Sfu1 was assayed in an iron-replete YPD medium for growth, and we compared each *sfu1*Δ/Δ mutant and its respective wild type. The *sfu1*Δ/Δ mutants showed upregulated iron acquisition genes in all strains, perhaps as a result of the upregulation of *SEF1* expression and the derepression due to *sfu1*Δ/Δ mutation (Fig. 3c; Table S2). The overall impact of Sfu1 on iron-related genes was fairly uniform among strains.

To quantify gene expression variation, we examined the SD of expression changes in *sef1*Δ/Δ vs wild-type comparisons. We used the Log_2_ fold change for each Sef1 direct target gene to calculate an SD across the five strains (Table S2, tab E) and present the results graphically (Fig. 3d). For the 36 Sef1 direct targets assayed by Nanostring, the mean SD was 0.38 (Fig. 3d). To provide a context for interpretation, we conducted the same calculation with Nanostring data for 81 direct Efg1 targets among the same five wild types and their respective *efg1*Δ/Δ mutants, using data from Huang et al. (12). For the Efg1 direct targets, the mean SD was 0.59 (Table S2, tab E; Fig. 3d). Because differences in SD might simply reflect the magnitude of upregulation or downregulation, we also compared only genes with a Log_2_ fold change in the range of −5 to −0.5 in deletion mutant vs wild-type comparisons (Fig. 3e). With this constraint, the 26 Sef1 direct targets had a mean SD of 0.41; the 21 Efg1 direct targets had a mean SD of 0.84 (Fig. 3e). These results indicate that *sef1*Δ/Δ gene expression impact is more uniform among strains than *efg1*Δ/Δ gene expression impact under the inducing conditions employed.

### Genome-wide view of iron regulatory variation

Our view of iron regulatory variation above is based upon a limited number of genes, with gene selection skewed toward those with large expression changes or clear functional connections to iron homeostasis. We also sought genome-wide insight into the questions of variation and conformity of the iron limitation response among strains. To that end, we used RNA-Seq to characterize the response to low iron and the impact of *sef1*Δ/Δ defects in two strain backgrounds, SC5314 and P57055. We chose these backgrounds to detect variation because, as shown above, they differed in the sensitivity of *sef1*Δ/Δ mutants to caspofungin and in the extent of YPD + BPS-induction of hypha-associated genes.

We first characterized the low-iron response in each strain through RNA-Seq analysis of cells grown in YPD + BPS or YPD (Table S3). We initially focused on gene expression changes with conventional thresholds (adjusted *P*-value < 0.05, Log_2_ fold change > 1 or < −1). We detected differential expression of 639 genes with strain SC5314 and 398 genes with strain P57055 (Table S3, Fig S4a, and Fig S4b). The 281 common upregulated genes (YPD + BPS vs YPD) were enriched for functions in iron transport and carbohydrate transport; the 246 common downregulated genes were enriched for functions in biosynthetic processes and translation. These responses reinforce previous microarray results (5). They make sense in that iron limitation may cause an energy deficit; cells may offset the deficit through reduced biogenesis rates and overcome the deficit through increased uptake of iron and energy sources.

We also tested the correlation between low-iron responses in the two strains by comparing the fold-change of differentially expressed genes chosen solely for an adjusted *P*-value < 0.05 in both strains. Our rationale is that some functionally important gene expression responses may be heterogeneous or transient and thus may fall below a specific fold-change in one strain. This approach previously indicated that the gene expression impact of *hgc1*Δ/Δ and *efg1*Δ/Δ mutations in these strain backgrounds correlates well (*R*^2^ of 0.75 and 0.79, respectively), while that of an *nrg1*Δ/Δ mutation correlates poorly (*R*^2^ of 0.38) (15). Here, we found that the low-iron responses correlated well, with an *R*^2^ of 0.83 for 820 genes (Fig. S4c). It seems reasonable that a physiologically driven response critical for survival would be quite uniform between clinical isolates.

How uniform is the gene expression impact of Sef1? We again began with a comparison of Sef1-responsive genes in the two strains with conventional cutoffs (*sef1*Δ/Δ vs WT; adjusted *P*-value < 0.05, log fold change >1 or < −1; Table S4). We detected differential expression of 417 genes in SC5314 and 274 genes in P57055. The 82 common downregulated genes (Fig. S5a) were enriched for iron homeostasis and iron transport functions (Fig. S5b), as expected (5). The 77 common upregulated genes (Fig. S5c) were enriched for respiration and biosynthesis functions (Fig. S5d), as expected (5). However, one set of upregulated genes was unexpected: those with functions related to damaged DNA binding (Fig. S5d).

We tested the correlation between Sef1-responsive genes in the two strains by the *P*-value-only selection approach described above. Sef1 responsive genes in the two strain backgrounds correlated with an *R*^2^ = 0.78 (Fig. S5e). That level of conformity is as high as seen previously (15).

We were curious to see if there was a gene expression correlated to the difference in caspofungin sensitivity of the *sef1*Δ/Δ mutants. Six cell wall integrity-associated genes responded differently to the *sef1*Δ/Δ mutation in the two backgrounds (Fig. S6). These six genes (*CRH11*, *PHR1*, *HAC1*, *PGA13*, *HWP2*, and *RCA1*) have positive roles in growth in the presence of cell wall stressors (39–45). Three of the genes were downregulated only in the caspofungin-hypersensitive *sef1*Δ/Δ mutant of SC5314. The other three genes were upregulated less in the *sef1*Δ/Δ mutant of SC5314 than in P57055. These gene expression differences may contribute to the phenotypic differences in *sef1*Δ/Δ mutant cell wall integrity.

### Role of Sef1 in iron limitation-induced DNA damage

**Fig 4:**
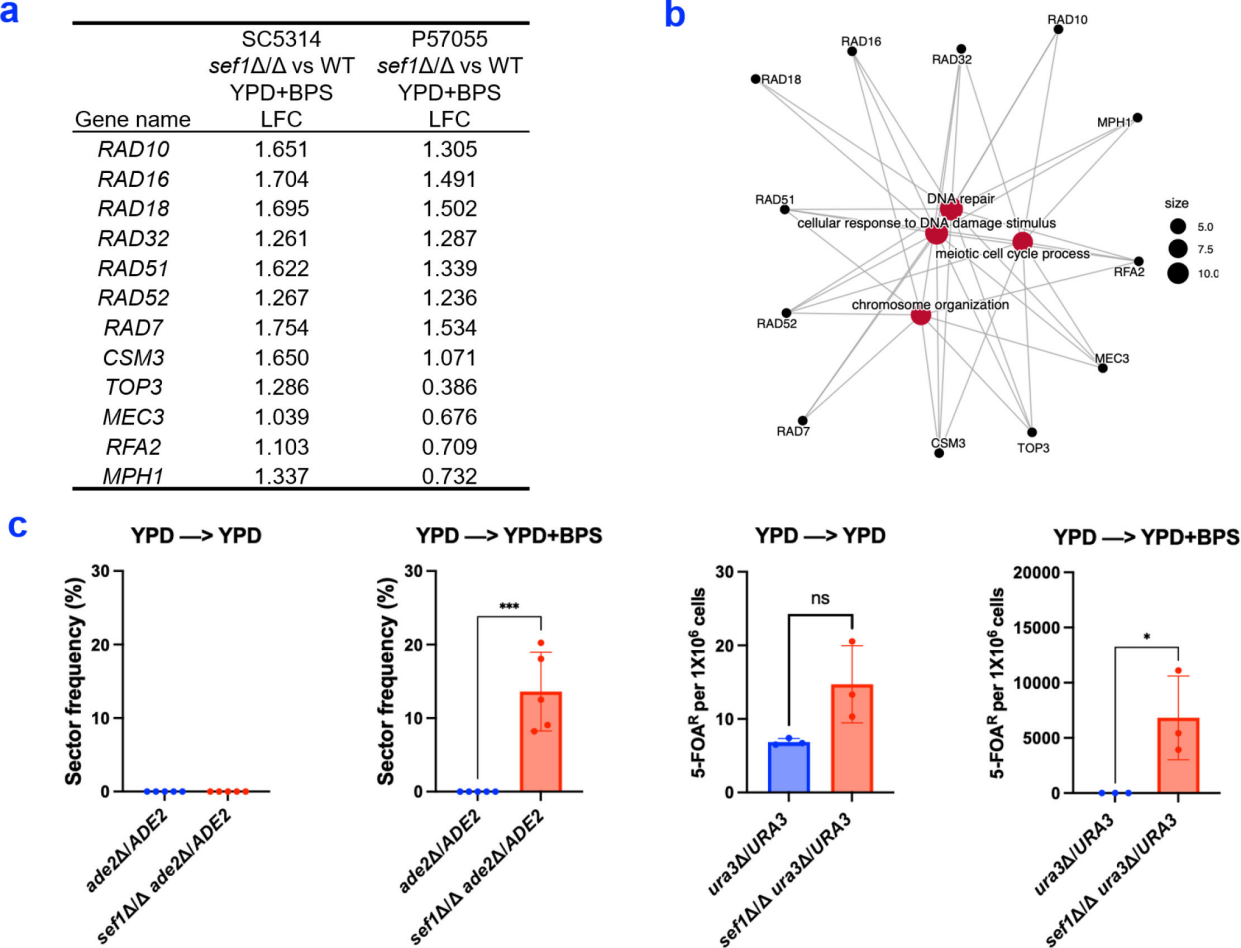
Impact of iron limitation on DNA repair. (**a**) A list of DNA damage and repair genes with significant upregulation (adjusted *P* < 0.05) and greater than twofold change in the *sef1*Δ/Δ vs wild-type comparison for at least one strain background. WT, wild-type strain. LFC, Log_2_ fold change. (**b**) A cnet plot graph generated by clusterProfiler (v4.8.1) in R by creating a GO term library using FungiDB [Candida albicans.Eupath.v65 (28)] with the R AnnotationForge package (26). Genes in this graph are associated with the GO terms for DNA repair, cellular response to DNA damage, chromosome organization, and mitotic cell cycle process. (**c**) Loss of heterozygosity assays. Quantification analysis of sector frequency (left) was conducted using strains SC5314 *ade2*Δ/*ADE2* and SC5314 *sef1*Δ/Δ *ade2*Δ/*ADE2*. Quantification analysis of 5-FOA resistant colonies per 1 × 10^6^ cells (right) was performed using strains SC5314 *ura3*Δ/*URA3* and SC5314 *sef1*Δ/Δ *ura3*Δ/*URA3*. Cells were grown overnight in YPD and then incubated on YPD + BPS plates before sampling for the emergence of recessive markers. Each dot in the graph represents an independent assay. Statistical analysis was performed using a *T* test. *P-value < 0.05 and ***P-value < 0.001.

Especially noteworthy was the upregulation of DNA repair genes in *sef1*Δ/Δ mutants of both strains under iron limitation (Fig. 4a and b). We considered the hypothesis that iron limitation may induce DNA damage in *sef1*Δ/Δ mutants. DNA damage may be repaired by crossovers to yield LOH, which can reveal recessive alleles. We assayed LOH with mutations in *ADE2* and *URA3* (Fig. S7).

LOH events in *ade2*Δ/*ADE2* heterozygotes can be estimated from the presence of red sectors in colonies (*ade2*Δ/Δ). A sector frequency of 13.6% was observed in *sef1*Δ/Δ *ade2*Δ/*ADE2* cells grown on YPD + BPS_100 μM_ (Fig. 4c) but not in otherwise wild-type *ade2*Δ/*ADE2* cells. LOH events in *ura3*Δ/*URA3* heterozygotes can be estimated from the emergence of 5-FOA-resistant colonies (*ura3*Δ/*ura3*Δ). A 5-FOA-resistant colony frequency of ~7,000 per 10^6^ cells was observed in *sef1*Δ/Δ *ura3*Δ/*URA3* cells grown on YPD + BPS_100 μM_ (Fig. 4c) but not in otherwise wild-type *ura3*Δ/*URA3* cells. For both loci, the apparent LOH events depended upon iron limitation imposed by BPS addition to the YPD growth medium (Fig. 4c). These results argue that Sef1 is required for genome integrity during iron limitation.

The connection between iron limitation and genome instability was first made, to our knowledge, with an *S. cerevisiae zim17* mutant, which is defective in iron-sulfur cluster assembly (46, 47). The mutant presented elevated rates of recombination and mutation (46). A more recent study of the fungal pathogen *Cryptococcus neoformans* showed that iron limitation of a wild-type strain caused upregulation of multiple double-strand break repair genes (48). Moreover, a *C. neoformans* mutant in iron homeostasis regulator Grx4 was found to be hypersensitive to DNA-damaging treatments (48). In these cases, as for the *C. albicans sef1*Δ/Δ mutant described here, DNA damage during iron limitation may result from defective activities of iron-dependent DNA replication and repair proteins, including DNA polymerases/primases, DNA helicases, and ribonucleotide reductase (49, 50).

## Data Availability

RNA-Seq data are available through the NCBI under BioProject ID PRJNA1079250.

## SUPPLEMENTAL MATERIAL

Supplemental figures msphere.00372-24-s0001.pdf Fig. S1-S7.

Table S1 msphere.00372-24-s0002.xlsx Strains, primers, plasmids.

Table S2 msphere.00372-24-s0003.xlsx Nanostring data and associated calculations.

Table S3 msphere.00372-24-s0004.xlsx RNA-Seq data.

ASM does not own the copyrights to Supplemental Material that may be linked to, or accessed through, an article. The authors have granted ASM a non-exclusive, world-wide license to publish the Supplemental Material files. Please contact the corresponding author directly for reuse.
