# Supplementary material for: Strain variation in the Candida albicans iron limitation response
Source: mSphere. 2024 Jul 9;9(7):e00372-24. doi: 10.1128/msphere.00372-24 (PMC11288005; doi:10.1128/msphere.00372-24)

Figure S1. Growth property under iron limitation with sole iron source. Five *Candida albicans* wild-type strains and their respective *sef1*Δ/Δ mutants were assayed for growth property in 5 media including SD pH 7.0, SD pH 7.0+BPS, SD pH 7.0+BPS+Ferric chloride, SD pH 7.0+BPS+hemin, SD pH 7.0+BPS+hemoglobin, SD pH 7.0+BPS+ferritin, and SD pH 7.0+BPS+transferrin. Concentrations of BPS and iron sources were added as indicated. Strains were grown in SD medium at 30 °C for 16 hours with shaking. Cells were harvested, washed by H2O, and then diluted to OD_{600}~3 in H_2O. Serial dilutions of 5^{-3} to 5^{-7} were spotted in indicated agar plates. Plates were incubated at 37 °C; images were taken at 72 hours. We estimate iron concentrations as follows. Hemin contains 1 iron/molecule; 50 uM of Hemin corresponds to 50 uM iron. Hemoglobin contains 4 iron/molecule and has a MW~64.5 kDa; 20 ug/ml of hemoglobin corresponds to 1.24 uM iron. Ferritin contains up to 4,500 Fe^{3+} per molecule and has MW~480 kDa; 20 ug/ml of ferritin corresponds to maximal 187.5 uM iron. Transferrin contains 2 iron/molecule and has a MW~80 kDa; 20 ug/ml of transferrin corresponds to 0.5 uM iron.

Figure S1

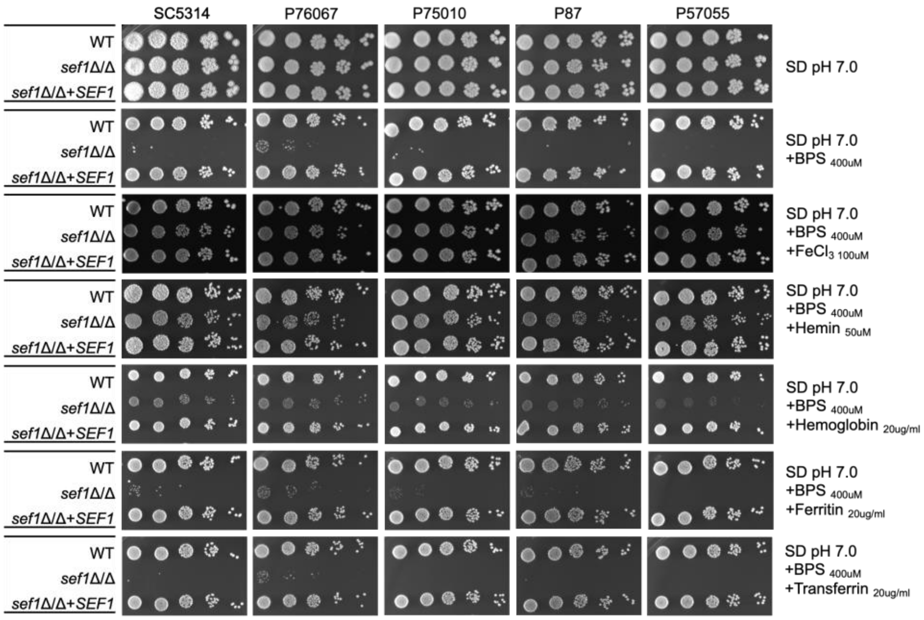

Figure S2. Cell wall integrity phenotypes. Five wild-type strains of *Candida albicans* (SC5314, P87, P75010, P76067 and P57055) and their respective *sef1*Δ/Δ derivatives were assayed for cell wall integrity with caspofungin in YPD solid media. Precultures were grown in YPD medium at 30 °C for 16 hours with shaking. Cells were harvested, washed with H_2O, and then diluted to OD_{600}~3 in H_2O. Serial dilutions of 5^{-3} to 5^{-7} were spotted in indicated agar plates. Caspofungin concentrations were 100 ng/ml (SC5314 strain set), 125 ng/ml (P87, P75010, P76067 and p57055 strain sets). Plates were incubated at 37 °C; images were taken at 72 hours.

Figure S2

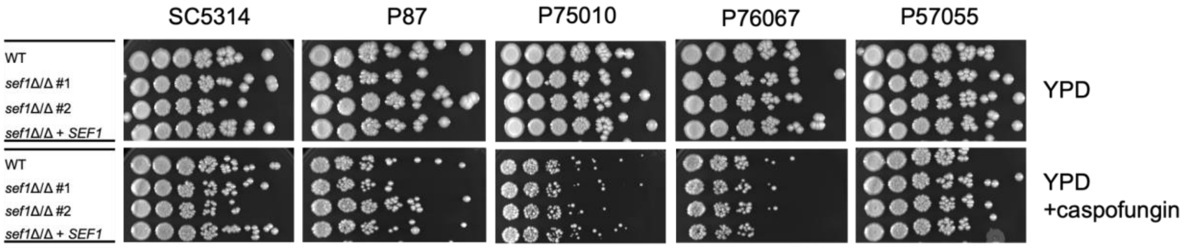

Figure S3. Correlation of hypha-associated gene expression and biofilm formation ability in a low-iron medium. We measured expression of 8 hypha-associated genes in response to iron limitation (YPD+BPS) by Nanostring, and present Log2 fold change for YPD vs YPD+BPS in the table (left panel). The results are condensed into the mean Log2 fold change of the 8 hypha-associated genes for each strain. We compared that mean value to biofilm depth in RPMI+FBS medium, which is low in iron, as determined by Huang et al. 2018, in the graph (right panel).

Reference: Huang MY, Woolford CA, May G, McManus CJ, Mitchell AP (2019) Circuit diversification in a biofilm regulatory network. PLoS Pathog 15(5): e1007787. https://doi.org/10.1371/journal.ppat.1007787

Figure S3

	LFC (Log2 Fold Change)				
**Gene Name**	**SC5314 WT YPD with BPS**	**P76067 WT YPD with BPS**	**P57055 WT YPD with BPS**	**P87 WT YPD with BPS**	**P75010 WT YPD with BPS**
ALS3	4.10	2.65	1.59	3.01	0.76
BCR1	0.28	0.21	0.23	0.13	0.22
BRG1	1.59	1.43	0.93	0.22	1.26
ECE1	5.50	3.68	2.69	4.99	0.54
HGC1	2.54	1.68	0.64	1.16	0.85
HWP1	4.27	2.35	2.82	3.89	0.61
HYR1	5.11	3.26	0.96	-0.33	0.68
IHD1	2.60	1.48	0.92	1.00	0.74
UME6	2.05	1.61	0.72	0.45	0.66
**Mean LFC**	3.11	2.04	1.28	1.61	0.70
**Biofilm depth in RPMI+FBS (um)**	**242**	**316**	**134**	**167**	**58**

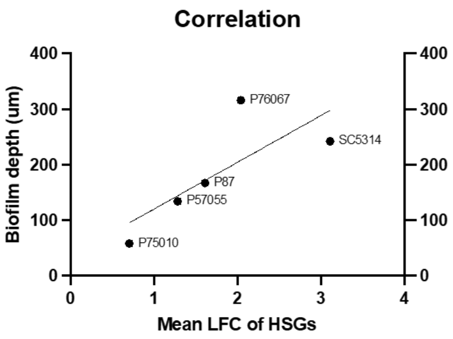

Figure S4. Low iron response. Global expression was assayed using RNA-Seq. Three biological replicates were analyzed for SC5314 and P57055 wild-type clinical isolates from cells grown in YPD with and without 500 μM of BPS for 4 h at 37 °C. (a, b) Venn diagrams depict the genes upregulated (a) or downregulated (b) with >2-fold change and an adjusted P-value <0.05 in YPD+BPS vs YPD of SC5314 and P57055. (c) Correlation analysis of low iron response. A total of 820 genes showed differential expression changes with an adjusted P-value <0.05 in both SC5314 and P57055. The X and Y axes represent wild-type YPD+BPS vs YPD Log_2 fold change for SC5314 and P57055, respectively. The correlation is represented by an R^2=0.83.

Figure S4

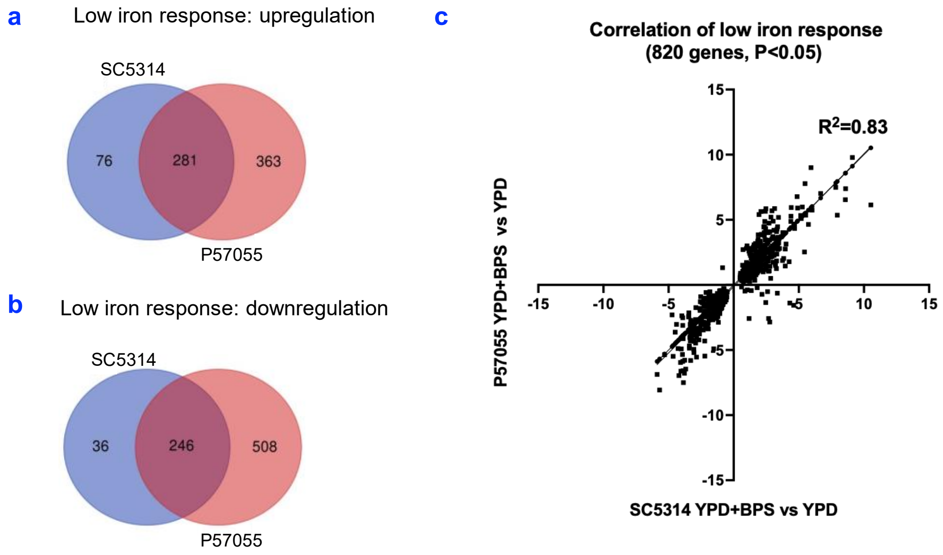

Figure S5. Gene expression analysis of *sef1*Δ/Δ response. Global expression was assayed using RNA-Seq. Three biological replicates were analyzed for two *sef1*Δ/Δ mutants and respective wild-type strains SC5314 and P57055 from cells grown in YPD with 500 μM of BPS for 4 h at 37 °C. (a, c) Venn diagrams depict the genes dependent upon Sef1 that are downregulated (a) or upregulated (c) with >2-fold change and an adjusted P-value <0.05 in both strain backgrounds. (b, d) GO enrichment analysis using clusterProfiler for genes downregulated (b) or upregulated (d) by the *sef1*Δ/Δ mutation in both strain backgrounds (26). The dot represents the number of genes in each category and categories with a P-value <0.05 were considered significant. (e) Correlation analysis of *sef1*Δ/Δ response. A total of 1335 Sef1 responsive genes commonly showed differential expression changes with an adjusted P-value <0.05 in both strain backgrounds. The X and Y axes represent *sef1*Δ/Δ vs wild type Log_2 fold change for SC5314 and P57055, respectively. The correlation is represented by an R^2=0.78.

Figure S5

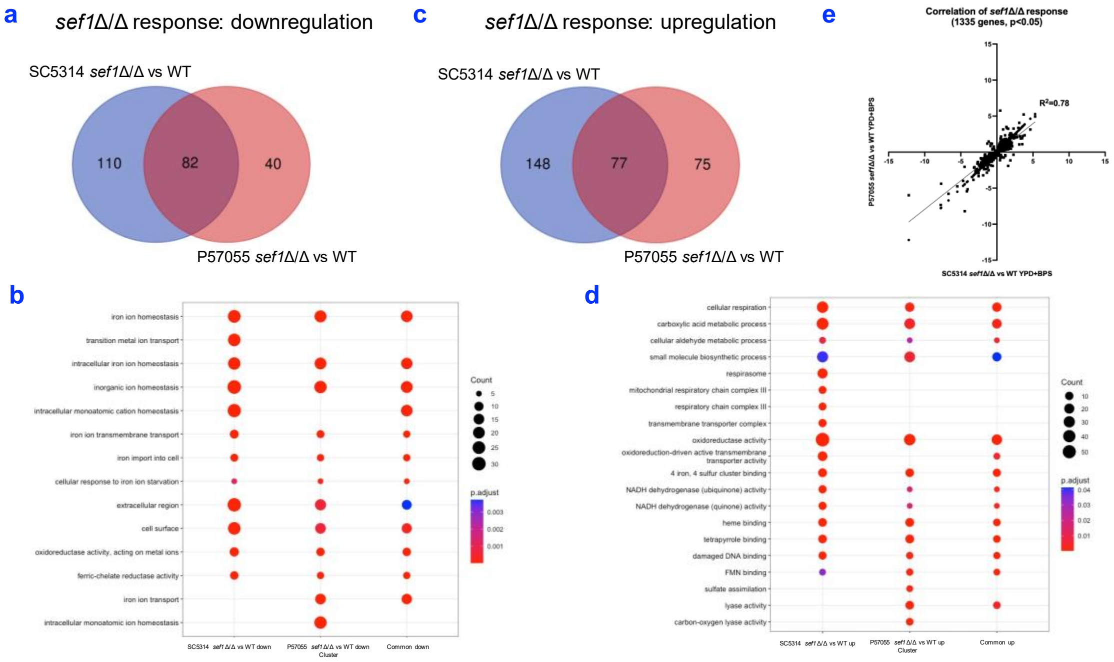

Figure S6. Cell wall integrity associated genes. Global expression was assayed using RNA-Seq. Three biological replicates were analyzed for two *sef1*Δ/Δ mutants and respective wild-type strains SC5314 and P57055 from cells grown in YPD with 500 μM of BPS for 4 h at 37 °C.

Figure S6

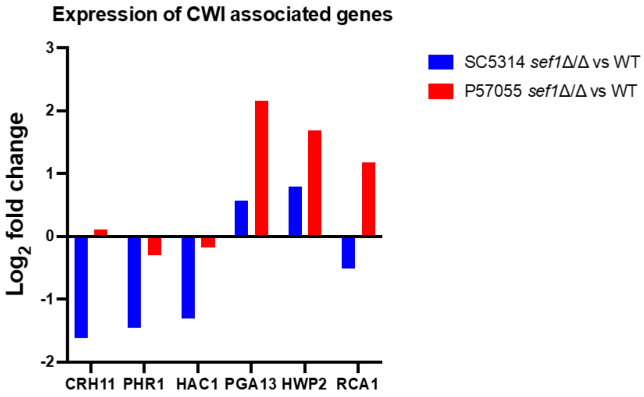

Figure S7. Workflows of LOH assays. LOH was assayed with mutations of *ADE2* (above) and *URA3* (below). Day 1, a fresh single colony was inoculated into fresh YPD medium and cultured at 30 °C overnight with rotation. Day 2, cells from overnight culture was inoculated to fresh YPD medium with an initial inoculum of OD_{600}~ 0.05 and grew for 24 hours at 30 °C with rotation. Day 3, cells were harvested and washed once by H_2O. Cell suspension was then diluted in H_2O to an OD_{600} of 3.0. After serial dilution, 100 µl of 10^{-4} dilution was plated on YPD and YPD+$BPS_{100µM}$, respectively. Plates were incubated at 37 °C and colony-formation-units (CFU) were counted when colonies grew to appropriate size. Day 12, colonies grown in YPD and YPD+$BPS_{100µM}$ agar plates were then stamped to fresh YPD plates and grew at 30 °C for another 5 to 7 days. Day 17-19, colonies with sectors formed were counted and calculated for frequency to total CFUs. For LOH assay of *URA3* mutation, cells were treated similarly as mentioned above from Day 1 to 3. Day 12, cells on YPD and YPD+$BPS_{100µM}$ agar plates were harvested and resuspended in H_2O. Then, cells were diluted in H_2O to an OD_{600} of 3.0, and 100 µl of 10^0 to 10^{-4} serial dilutions were plated on the agar plates CSM and CSM+uridine+5-FOA, respectively. Day 14 to 15, plates were incubated at 30 °C and CFU was counted.

Figure S7

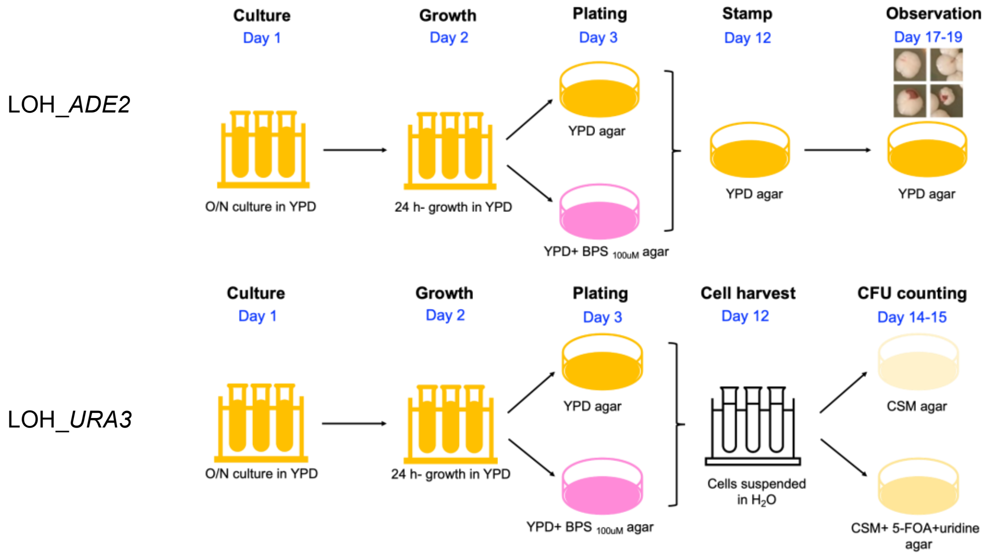

Supplement: Supplemental figures — Fig. S1-S7. [file msphere.00372-24-s0001.pdf]
